# Using plasma cell-free mRNA to profile immune response and myocardial damage in immune checkpoint inhibitor–induced myocarditis

**DOI:** 10.1172/JCI188817

**Published:** 2025-08-15

**Authors:** Alireza Raissadati, Xuanyu Zhou, Harrison Chou, Yuhsin Vivian Huang, Shaheen Khatua, Yin Sun, Anne Xu, Sharon Loa, Arturo Hernandez, Han Zhu, Sean M. Wu

**Affiliations:** 1 Stanford Cardiovascular Institute, Stanford University School of Medicine, Stanford, California, USA.; 2 Department of Pediatrics, Stanford University, Stanford, California, USA.; 3 Department of Biomedical Sciences, and; 4 Division of Cardiovascular Medicine, Department of Medicine, Stanford University School of Medicine, Stanford, California, USA.

**Keywords:** Cardiology, Immunology, Autoimmune diseases, Diagnostics, Molecular diagnosis

## Abstract

Plasma cell-free mRNA provides tissue-specific transcriptional profiling, precisely capturing cardiac damage and immune responses in immunotherapy-induced myocarditis.

**To the Editor:** Immune checkpoint inhibitors (ICIs) have revolutionized cancer therapy, but their immune-related adverse events (irAEs) remain challenging. ICI myocarditis (ICI-m) remains a rare but lethal irAE, with a mortality of up to 40% (1). Recent studies indicate clonally expanded cytotoxic terminally differentiated effector memory CD8+ T cells reexpressing CD45RA (Temra CD8+ T cells) as the driver of ICI-m, manifesting as lymphocyte- and macrophage-rich myocardial infiltrates on histopathology (2–4). Diagnosis with cross-sectional imaging, biochemical markers, and endomyocardial biopsy is either nonspecific, insensitive, or highly invasive, highlighting the urgent need for more disease-specific biomarkers (5). Plasma cell-free mRNA (cf-mRNA) is a promising diagnostic tool that can be used as a “liquid biopsy,” capturing tissue-specific transcriptomic profiles (6). Existing cf-mRNA analyses lack the cell-type granularity required for true disease-specific cf-mRNA mapping (6). We developed a cf-mRNA platform for high-resolution assessment of tissue damage and activity and validated our platform in a cohort of patients with ICI-m (Figure 1A). Our approach captured both the disease-specific Temra CD8+ T cell response and consequent cardiomyocyte damage, demonstrating the potential of cf-mRNA disease profiling of ICI-m.

We analyzed 22 ICI-treated patients with cancer in the discovery cohort: 5 group A patients with ICI but no irAEs, 7 group B patients with ICI and only extracardiac irAEs, and 10 group C patients with myocarditis per the Bonaca and International Cardio-Oncology Society criteria (see supplemental materials; supplemental material available online with this article; https://doi.org/10.1172/JCI188817DS1) with or without concomitant extracardiac irAEs after sample quality filtering (Supplemental Table 1). Six separate group C patients were included in the validation cohort along with 30 individuals acting as healthy controls from a previous publication (see supplemental materials) for external comparison. Our cf-mRNA pipeline yielded consistent amounts of high-quality cf-RNA across all groups (Figure 1, B and C), with a characteristic scattered size distribution, exon predominance, and protein-coding majority (Supplemental Figure 1, A–C). Transcriptomic analysis of ratio-batch corrected gene counts revealed 10,718 differentially expressed genes (DEGs) between ICI-treated patients with cancer and individuals acting as healthy controls (Figure 1D). Unsupervised nonnegative matrix factorization analysis of the upregulated protein-coding DEGs produced two main clusters: one enriched for immune response pathways, including a CCL5-driven processes, and another for oncogenic processes through Ingenuity Pathway Analysis (IPA; Supplemental Figure 2, A and B).

To capture cardiac-specific signals, we compared group C and A patients, identifying 1,222 DEGs (Figure 1E) with emergence of cardiac-related IPA pathways, particularly cardiac conduction and cardiomyocyte contraction (Figure 1F). The previously highlighted myocardial antigen MYH6 and disease-specific cytokine CCL5 were elevated in group C versus group A patients (Figure 1G) (3, 4).

We mapped cf-mRNA to cell types using our previously published single-cell RNA-Seq data of circulating PBMCs from ICI-treated patients with cancer (3) and the human heart atlas (see supplemental materials). CD8+ T cell, specifically Temra CD8+ T cell, signatures were significantly elevated in group C patients compared with A and B patients (Figure 1, H and I). External validation using the Tabula Sapiens blood immune cell dataset (see supplemental materials) corroborated these findings, showing elevated CD8+ effector T cell signatures in patients with ICI-m. Cardiac cf-mRNA profiling demonstrated increased signature scores across cardiomyocytes and conduction system cells in group C patients (Figure 1, J and K, and Supplemental Figure 2, C and D).

Through cross-examination of DEGs from group C versus A, C versus B, and B versus A, we developed a 6-gene classifier (3 from the cardiac gene panel: ZNF385B, CADM2, AQP7; 3 from the immune: B2M, IFITM2, CCL5) for diagnosing ICI-m (Figure 1L). The classifier outperformed an unsupervised 37-gene panel in distinguishing group C from group A and B patients in both the discovery (Figure 1M and Supplemental Figure 3A) and validation cohorts (Figure 1N and Supplemental Figure 3B). Our classifier also showed superior specificity in differentiating patients with ICI-m from individuals acting as healthy controls compared with its performance with other patient groups (Supplemental Figure 3C).

Plasma cf-mRNA profiling distinguishes ICI-m from other irAEs through precise cellular mapping. Unlike traditional biomarkers, such as proteins, cfDNA, and miRNAs, which lack cell-type resolution, cf-mRNA enables detailed tissue profiling in a cell-type-specific fashion. Leveraging this, we developed a targeted 6-gene classifier that outperformed conventional unsupervised analysis approach to identify ICI-m among ICI-treated patients with cancer. While these findings establish cf-mRNA as a promising noninvasive liquid biopsy tool, larger validation cohorts and standardized processing protocols are needed to advance this technology toward clinical implementation and explore its potential in diagnosing other inflammatory conditions.

## Supplementary Material

Supplemental data

Supporting data values

## Figures and Tables

**Figure 1:**
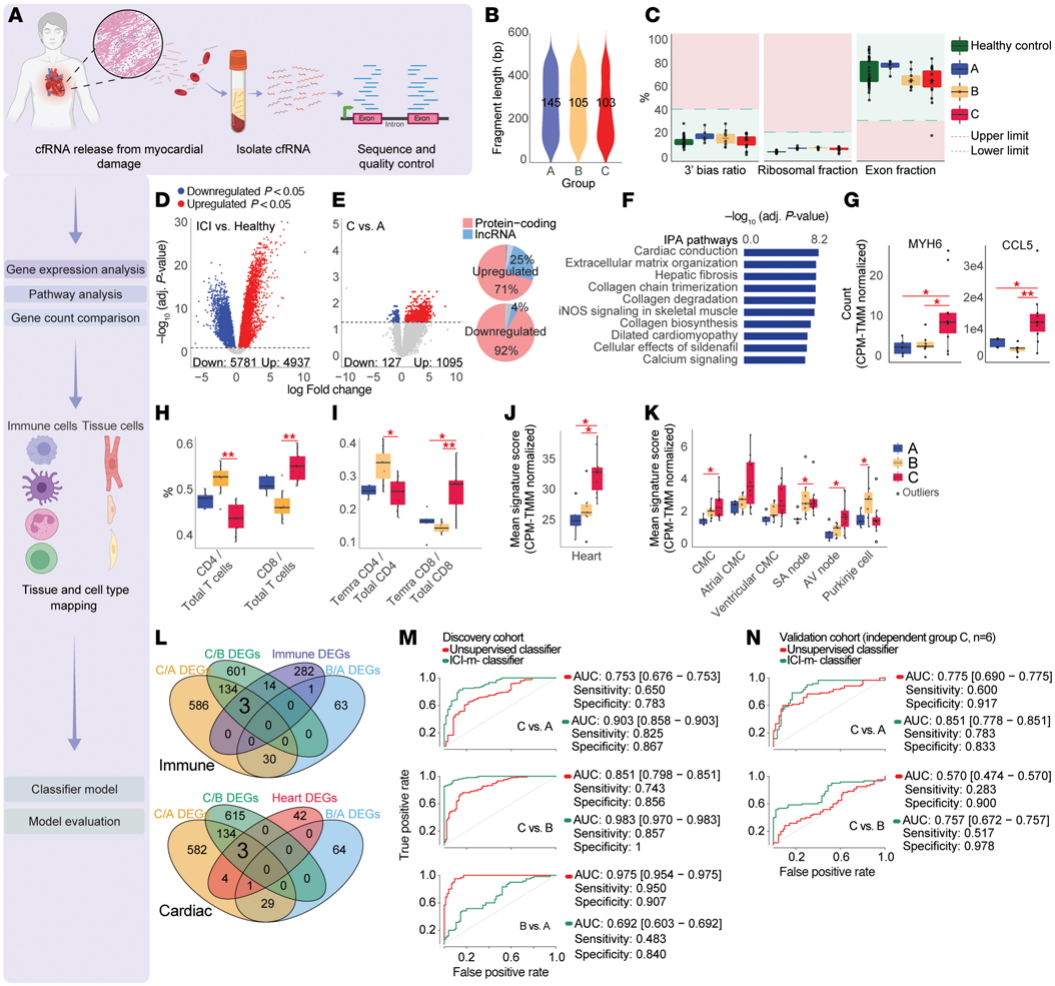
Plasma cf-mRNA profiling distinguishes patient populations and disease pathways. (**A**) Schematic of experimental and computational pipeline. (**B**) cf-RNA yields and average fragment lengths from groups A, B, and C. (**C**) QC pipeline for screening degraded samples (3′ bias), ribosomal content, and DNA contamination. (**D**) Volcano plot:differentially expressed genes between ICI-treated patients with cancer (A+B+C, *n* = 22) and individuals acting as healthy controls (*n* = 30) (Benjamini-Hochberg –adjusted [BH-adjusted] *P* < 0.05), showing a higher number of differential genes. (**E**) Volcano plot: group C versus A (BH-adjusted *P* < 0.05) for cardiac pathways, with gene-type charts showing majority protein-coding genes. (**F**) IPA pathway analysis of upregulated differentially expressed genes in group C versus A patients. (**G**) Box plots: counts per million–trimmed mean of M values–normalized (CPM-TMM-normalized) counts of MYH6 and CCL5 cf-mRNA in group C versus A and B. (**H** and **I**) Box plots: CD4^+^/CD8^+^ T cell and Temra CD4^+^/CD8^+^ T cell proportions from single-cell RNA-Seq gene panels of ICI-treated patient PBMCs (3). (**J** and **K**) Heart and cardiomyocyte signature scores in group C versus A and B. *P < 0.05, **P < 0.01 by Mann-Whitney *U* test comparing groups. (**L**) Venn diagram: 6 genes (3 cardiac, 3 immune) were identified from C versus A and C versus B DEGs but not overlapping with B versus A DEGs to constitute an ICI-m–specific diagnostic panel. (**M**) ROC curves with metrics for C versus A (*n* = 10 vs 5), C versus B (*n* = 10 vs 7), and B versus A (*n* = 7 vs 5) using unsupervised panel (red) and ICI-m classifier (green). (**N**) ROC validation: both panels on separate group C (*n* = 6) versus groups A (*n* = 5) and B (*n* = 7).

## Version 1 08/15/2025

Electronic publication
