# Supplementary material for: Using plasma cell-free mRNA to profile immune response and myocardial damage in immune checkpoint inhibitor–induced myocarditis
Source: J Clin Invest. 2025 Aug 15;135(16):e188817. doi: 10.1172/JCI188817 (PMC12352903; doi:10.1172/JCI188817)
Supplement: Supplemental data [file jci-135-188817-s161.pdf]

Using plasma cell-free mRNA to profile immune response and myocardial damage in immune checkpoint inhibitor-induced myocarditis

Alireza Raissadati[1,2], Xuanyu Zhou[1,3], Harrison Chou[1], Yuhsin Vivian Huang[1], Shaheen Khatua[1], Yin Sun[1], Anne Xu[1], Sharon Loa[1], Arturo Hernandez[1], Han Zhu[1,4,*], Sean M. Wu[1,4,*]

[1]Stanford Cardiovascular Institute, Stanford University School of Medicine, Stanford, CA, United States

[2]Division of Cardiology, Department of Pediatrics, Stanford University School of Medicine, Stanford, CA, United States

[3]Department of Biomedical Sciences, Stanford University School of Medicine, Stanford, CA, United States

[4]Division of Cardiovascular Medicine, Department of Medicine, Stanford School of Medicine, Stanford, CA 94305

[*]Shared corresponding authorship. Emails: hanzhu@stanford.edu, smwu@stanford.edu

Conflict of interest

The authors declare no competing interest regarding the work described in this paper.

SUPPLEMENTAL METHODS

All individual graphed datapoints are supplied at a sample-level in the Supporting data values spreadsheet.

Sex as a biological variable

We used samples from both female and male subjects in all patient cohorts and includes sex as a covariate in our analyses.

Definition of ICI-m

ICI myocarditis was diagnosed using Bonaca and International Cardio-Oncology Society criteria incorporating clinical data including elevated cardiac biomarkers (troponin I>99th percentile of the general population), clinical syndrome, echocardiography, negative coronary work-up with computed tomography, imaging diagnosis (magnetic resonance imaging and/or PET), and/or endomyocardial biopsy when available (1, 2).

Plasma processing

We obtained peripheral blood samples after written informed consent/assent from all participants after clinical diagnosis of ICI-m and prior to invasive procedures. Plasma was isolated from EDTA vacutainer blood tubes by density gradient separation with Ficoll-Paque PLUS (GE Healthcare Biosciences, Uppsala, Sweden) within 6 hours of collection. Blood was slowly layered on 5 mL of Ficoll-Paque PLUS in a 15 mL conical tube by pipetting carefully down the side of the tube with a transfer pipette. Tubes were centrifuged at 2000 rpm for 20 minutes with brakes off. PBMCs were aspirated from the density. To ensure consistent processing conditions, all plasma samples

were separately placed in cryopreservation tubes and preserved in liquid nitrogen and later processed simultaneously for cf-mRNA isolation and sequencing, thus minimizing batch effects.

cfRNA isolation

cfRNA was isolated from 900-1000ul of plasma using the Norgen MIDI plasma/serum RNA purification kit (SKU56200, Norgen) per kit instructions. Samples were DNase treated on-column for 15min at 27C using the Norgen RNase-free DNase I kit (SKU25710, Norgen) and reconstituted into 30ul. Isolated cfRNA concentrations were estimated using the Bioanalyzer RNA 6000 Pico Kit (5067-1513, Agilent) per manufacturer instructions, with the remainder stored in -80C until library preparation.

Sequencing library preparation

Libraries were prepared for sequencing with the SMARTer Stranded Total RNAseq Kit v3 – Pico Input Mammalian Components (634485, Takara) from 8ul of cfRNA according to the manufacturer's instructions. Samples were barcoded using the SMARTer RNA Unique Dual Index Kit (634451, Takara), and then pooled in an equimolar manner and sequenced on Illumina's NovaSeq platform (2 x 150bp) to a mean depth of 25GB. Library concentrations were estimated from 1ul of 1:1 diluted elution using the Bioanalyzer High Sensitivity DNA assay (5067-4626, Agilent) per manufacturer instructions, with the remainder stored in -20 until sequencing.

Bioinformatic processing

Raw sequencing reads were trimmed using TrimGalore (v.0.6.10) with automated adapter sequence identification. Duplicate reads were removed based on unique molecular indices with

umi-tools (v.1.1.4). Reads were mapped to the hg38 genome using Star (v.2.7.10) and v.45 of the gencode gene annotation files. Read statistics were estimated using FastQC (v.0.12.1) after each processing step. Sequence composition was assessed using RSeQC (v. 5.0.1) and gene counts generated from all mapped reads using Featurecounts from the Rsubread R package (v.2.12.3).

Sample quality filtering

Samples were filtered with custom code quantifying RNA degradation, DNA contamination, and proportion of ribosomal RNA, per previously published data (3). Briefly, RNA degradation was measured as the proportion of genes for which all reads mapped exclusively to the 3'-most exon (3' bias fraction, cutoff ≥0.4). DNA contamination was evaluated as the ratio of introns to exons (cutoff ≤0.3), and Ribosomal RNA fraction was determined as the proportion of reads mapping to the ribosome (region GL00220.1:105,424–118,780, hg38) relative to all reads (SAMtools view, cutoff ≥0.2). Finally, we excluded any samples with a total gene count of <200,000. Samples that failed any of these metrics were censored.

Gene filtering

To maintain focus on biologically relevant genes, we only included features with ≥1 count across ≥50% of samples. With this approach, 18,590 genes remained after filtering (44,651 genes excluded).

Fragment and gene type profiling

Fragment sizes were estimated as the insert size between two paired reads and plotted as a percentage of total fragment counts. Fragment gene type was determined using v.45 of the gencode

gene annotations and only protein-coding genes included in all downstream analyses (14,876 protein-coding genes).

Batch correction of healthy control data

To correct for batch effects between the healthy controls (Roskams-Hieter et al. dataset) and ICI cancer samples, we employed a ratio-based normalization approach using the Roskams-Hieter dataset as the reference group (4). First, we calculated the average expression of the reference sample within each batch. Next, we computed the ratio-based expression values by dividing the gene counts in each sample by the corresponding reference sample average. This normalization step effectively adjusted the gene expression levels to account for batch-specific differences. PCA plots and UMAPs before and after batch correction were inspected to ensure adequate batch correction.

Differential gene expression analysis

For comparing the batch corrected ICI cancer group (combined groups A-C, n=22) to healthy controls, we performed differential expression analysis in R using edgeR (v.3.40.2) with age and sex as covariates. For comparing groups A, B, and C, we performed differential expression analysis in R using DSeQ2 (v.1.42.1) including age and sex as covariates in the design. In both methods, differentially expressed genes (DEG) were identified using Benjamini-Hochberg multiple hypothesis adjustment at a significance threshold of 0.05.

Functional enrichment analysis

Gene Ontology (GO) pathway analysis and ingenuity Pathway Analysis (IPA) were performed separately for up- and downregulated DEGs and cardiac-specific pathways isolated for further inspection. Results are presented as horizontal bar graphs with values representing negative log10 of the BH-adjusted p-value of each biological pathway.

Non-negative matrix factorization

We performed non-negative matrix factorization (NMF) analysis on significantly upregulated DEGs (BH-adjusted $p<0.05$) from the batch-corrected data using the NMF package in R (v.0.27). The optimal factorization rank was estimated using the brunet method with 50 runs. GO pathway and IPA enrichment analysis was performed for each cluster using the clusterProfiler package in R (v.4.6.2), and the top 10 pathways per cluster based on adjusted p-values were extracted.

Defining cell-type- and tissue-specific gene profiles

We defined cell type-specific gene expression profiles using the human heart scRNAseq dataset by Litviňuková et al and our previously published scRNAseq dataset from the circulating PBMCs from our ICI cohort including all current A, B, and C patients (5, 6). In addition, we used the healthy blood immune cell scRNAseq dataset from Tabula Sapiens as a control for the ICI-treated PBMC phenotypes (7). The raw gene expression counts were normalized to a total count of 10,000 per cell and log-transformed using Scanpy (v.1.9.3). Differential expression analysis was then performed for each cell type using the Wilcoxon rank-sum test, followed by multiple testing correction using the Benjamini-Hochberg method. Differentially expressed genes were filtered based on a minimum fold change of 1.5, a minimum fraction of 0.2 expressing cells in the target group, and a maximum fraction of 0.5 expressing cells in the background groups. The top 20 DEGs

for each cell type were selected based on ascending adjusted p-values. Finally, we generated a unique set of DEGs for each cell type by assigning each gene to the cell type with the highest mean expression, ensuring a distinctive and non-overlapping gene profile. Stacked violin plots were inspected to ensure cell type specificity.

Defining signature score per cell type or tissue

The cell type signature score was defined as the sum of the logCPM and TMM-normalized values of all respective cell-type-specific genes within the sample as described previously (8). We first calculated the mean expression counts for each cell type and condition, stratified by tissue, using the log-transformed and normalized gene expression data. We then computed the log2 ratio of mean counts between the group of interest and control group for each cell type within each tissue for forest plots and the absolute signature scores for all three groups for comparison in box plots. To assess the range of the signature scores within each patient group, we calculated individual sample ratios for the condition of interest with a pseudocount of 1 within each tissue. The mean and standard deviation of these log2 ratios were determined for each cell type within each tissue for the forest plots, and for each box for the three-group comparison with box plots.

We used the non-parametric Mann-Whitney U test to identify statistically significant cell-type signature scores between conditions. Cell types with insufficient data variation, defined as having fewer than one sample in either condition, were excluded from the analysis. Cell types with a p-value < 0.05 were considered statistically significant. We generated forest plots displaying the mean log2 ratio of signature scores for each cell type within each tissue, with error bars representing the standard deviation. Individual sample ratios were overlaid on the forest plots as data points.

Conventional biomarker discovery using feature selection with machine learning

To represent conventional biomarker discovery, we employed a combination of feature selection and machine learning techniques to develop a classifier for diagnosing ICI-m. First, we merged all upregulated DEGs obtained from comparing C vs. A and C vs. B using a BH-adjusted p-value cutoff of $p<0.05$. We then applied Recursive Feature Elimination with Cross-Validation (RFECV) using a linear Support Vector Machine (SVM) as the estimator with repeated k-fold cross-validation, ultimately selecting the most informative genes for distinguishing group C from A and B in an unsupervised fashion.

Cell type mapping-derived ICI-m classifier

For our custom cell type mapping-derived ICI-m classifier, we first isolated all upregulated DEGs from C vs. A and C vs. B comparisons (n=134), ensuring none of the DEGs intersected with upregulated DEGs from B vs. C comparison. Next, we intersected the top 20 cell type-specific genes of all cell types from the Zhu et al. circulating PBMC scRNAseq dataset and of the cardiomyocytes from the Litviňuková et al. heart scRNAseq dataset with the 134 combined DEGs. We chose to only use cardiomyocytes from the heart scRNAseq dataset given the highest specificity to the heart and less probability of overlap with other extracardiac cellular phenotypes.

Determining cell types enriched in the classifiers

To determine the enrichment of cardiac and immune cells in the ICI-m classifier and unsupervised classifier, we intersected the cell type-specific genes of each scRNAseq dataset separately with each gene panel. For each cell type and tissue, we calculated the overlap between the DEGs in the gene panel and the corresponding specific gene set using a hypergeometric test with the Benjamini-

Hochberg method to adjust for multiple testing. Cell types and tissues with an adjusted p-value < 0.05 were considered significantly enriched in the upregulated DEGs. Results are presented as the proportion of genes specific to each cell significantly enriched in each classifier, plotted as a horizontal bar chart.

Performance metric analysis

We evaluated classifier performance using cross-validation and multiple metrics. The dataset was split into 80% training and 20% test sets. We used repeated 2-fold cross-validation with 15 repeats and the glmnet algorithm for training. Performance was assessed using the ROC curve, AUC, sensitivity, and specificity at the optimal cut-off point determined by Youden's index. We generated plots for the ROC curve, ROC values across model iterations, predicted probability distributions, and confusion matrix. All metrics and plots were generated using the caret (v.6.0-94) and ggplot2 (v.3.5.0) packages in R. The rigorous cross-validation scheme and evaluation of multiple metrics ensured a comprehensive assessment of the classifiers' performance and potential for accurate classification.

Statistics overview

We used two-tailed Mann-Whitney U test for all intergroup comparisons with a p-value of <0.05 considered statistically significant. Genes were considered statistically significantly differentially expressed based on a Benjamini-Hochberg-adjusted P value of <0.05. This threshold was chosen for statistical rigor while avoiding loss of biological signal capture due to overtly stringent criteria.

Study approval

Our study was approved by the institutional review board at Stanford, Palo Alto. All samples used in the study were obtained from patients consented for biobanking, gene sequencing, and sharing of their sequenced data in a de-identified manner outside of Stanford.

Data availability

All plasma cfRNA sequencing data presented in this study is stored as raw FASTQ sequencing files in the GEO database under the accession number GSE296680. cfRNA data for the healthy cohort (Roskams-Hieter B et al) can be accessed under the accession number GSE182824.

Acknowledgements

This study was funded by grants from the Burroughs Wellcome Fund and the pediatric scientist development program (PSDP), Oskari Huttunen Foundation, and Emil Aaltonen Foundation (to AR); from the NIH (1K08HL16140501, to HZ); and Additional Venture Foundation, Joan and Sanford I. Weill Scholar Fund, and the NSF RECODE grant (to SMW). The authors extend their gratitude towards the following people for allowing access to their patients for recruitment based on the Institutional Review Board–approved protocol in this article: Drs Alice Fan, Sukhimani Padda, Kavitha Ramchandran, Dimitrios Colevas, Sumit Shah, Maximilian Diehn, Michael B. Fowler, and Randall Vagelos. In addition, they thank the Stanford Cancer Center and Stanford Cardiovascular Research Institute.

References

1. Salem J-E, Manouchehri A, Moey M, et al. Cardiovascular toxicities associated with immune checkpoint inhibitors: an observational, retrospective, pharmacovigilance study. *The Lancet Oncology*. 2018;19:1579–1589.

2. Bonaca MP, Olenchock BA, Salem JE, et al. Myocarditis in the setting of cancer therapeutics: proposed case definitions for emerging clinical syndromes in cardio-oncology. *Circulation*. 2019;140:80-91.

3. Moufarrej MN, Wong RJ, Shaw GM, et al. Investigating Pregnancy and Its Complications Using Circulating Cell-Free RNA in Women's Blood During Gestation. *Front Pediatr*. 2020;8:605219.

4. Roskams-Hieter B, Kim HJ, Anur P, et al. Plasma cell-free RNA profiling distinguishes cancers from pre-malignant conditions in solid and hematologic malignancies. *npj Precis. Onc*. 2022;6:28.

5. Zhu H, Galdos FX, Lee D, et al. Identification of Pathogenic Immune Cell Subsets Associated With Checkpoint Inhibitor–Induced Myocarditis. *Circulation*. 2022;146:316–335.

6. Litviňuková M, Talavera-López C, Maatz H, et al. Cells of the adult human heart. *Nature*. 2020;588:466–472.

7. The Tabula Sapiens Consortium*, Jones RC, Karkanias J, et al. The Tabula Sapiens: A multiple-organ, single-cell transcriptomic atlas of humans. *Science*. 2022;376:eabl4896.

8. Robinson MD, Oshlack A. A scaling normalization method for differential expression analysis of RNA-seq data. *Genome Biol*. 2010;11:R25.

SUPPLEMENTARY MATERIAL

Supplemental Table 1 - Patient demographics.

	A (n=5)	B (n=7)	C (n=10)	Healthy Control (n=30)
**Age, (yrs, mean ±SD)**	70.5 (18.2)	61.3 (12.2)	76.8 (6.14)	52.9 (14.7)
**Sex, n (%)**				
Female	2 (40.0)	1 (14.3)	6 (60.0)	19 (63.3)
Male	3 (60.0)	6 (85.7)	4 (40.0)	11 (36.7.1)
**Race, n (%)**				
Asian	0 (0)	2 (28.6)	1 (10)	
White	3 (60.0)	5 (71.4)	9 (90.0)	
Other	2 (40)	0 (0)	0 (0)	
**Weight (kg, mean ±SD)**	89.1 (24.6)	80.9 (15.1)	69.8 (12.5)	
**BMI (mean, ±SD)**	30.4 (5.51)	26.3 (4.61)	24.9 (3.52)	
**Smoking Status n (%)**				
Former	1 (20.0)	2 (28.6)	2 (20.0)	
Never	4 (80.0)	4 (57.1)	8 (80.0)	
Every Day	0 (0)	1 (14.3)	0 (0)	
**Alcohol Use, n (%)**				
Yes	2 (40.0)	4 (57.1)	4 (40.0)	
No	3 (60.0)	3 (42.9)	6 (60.0)	
**Cancer Type, n (%)**				
Bladder	1 (20.0)	0 (0)	0 (0)	
Kidney	2 (40.0)	2 (28.6)	3 (30.0)	
Sarcoma	1 (20.0)	0 (0)	0 (0)	
Skin	1 (20.0)	1 (14.3)	0 (0)	
Head and Neck	0 (0)	1 (14.3)	0 (0)	
Lung	0 (0)	1 (14.3)	0 (0)	
Stomach	0 (0)	1 (14.3)	2 (20.0)	
Thyroid	0 (0)	1 (14.3)	0 (0)	
Brain	0 (0)	0 (0)	1 (10.0)	
Liver	0 (0)	0 (0)	1 (10.0)	
Prostate	0 (0)	0 (0)	1 (10.0)	
Uterine	0 (0)	0 (0)	2 (20.0)	
**Immunotherapy, n (%)**				
Pembrolizumab	5 (100)	3 (42.9)	5 (50.0)	
Nivolumab	2 (40.0)	4 (57.1)	3 (30.0)	
Cemiplimab	0 (0)	0 (0)	1 (10.0)	
Atezolizumab	0 (0)	1 (14.3)	0 (0)	
Ipilimumab	0 (0)	2 (28.6)	2 (20.0)	
Other	3 (60.0)	2 (28.6)	0 (0)	
**Inhibitor Type, n (%)**				
PD1	5 (100)	6 (85.7)	9 (90.0)	
PDL-1	0 (0)	1 (14.3)	0 (0)	
CTLA-4	0 (0)	1 (14.3)	2 (20.0)	
Chemotherapy	1 (20.0)	5 (71.4)	1 (10.0)	
Missing	0 (0)	0 (0)	2 (20.0)	
**IRAE, n (%)**				
Thyroiditis	0 (0)	2 (28.6)	1 (10.0)	
Dermatitis	0 (0)	4 (57.1)	0 (0)	
Myositis	0 (0)	1 (14.3)	2 (20.0)	
Gastritis	0 (0)	1 (14.3)	0 (0)	
Nephritis	0 (0)	1 (14.3)	0 (0)	
Other IRAE	0 (0)	4 (57.1)	3 (30.0)	
No IRAE	0 (0)	0 (0)	7 (70.0)	

SUPPLEMENTAL FIGURES

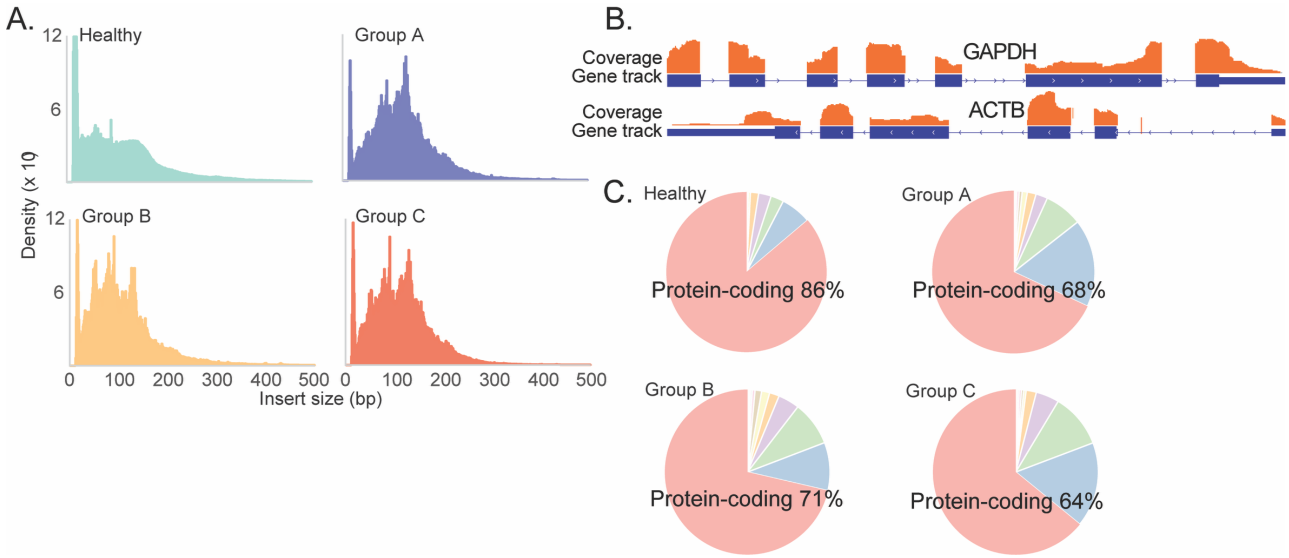

Supplemental Figure 1 – Our cf-RNA pipeline successfully captures protein-coding genes with minimal DNA contamination and background ribosomal RNA noise. A) Fragment profiles of all cf-RNA fragments of healthy controls (n=30) and all ICI cancer patients (A=5, B=7, C=10 patients per group) **B)** Coverage tracks of cf-RNA on GAPDH and ACTB confirming exonic capture. **C)** Gene type fractions of each patient group demonstrating majority protein coding genes in all patient groups. ICI, immune checkpoint-inhibitor; cf-RNA, cell-free total RNA; BP, base pairs.

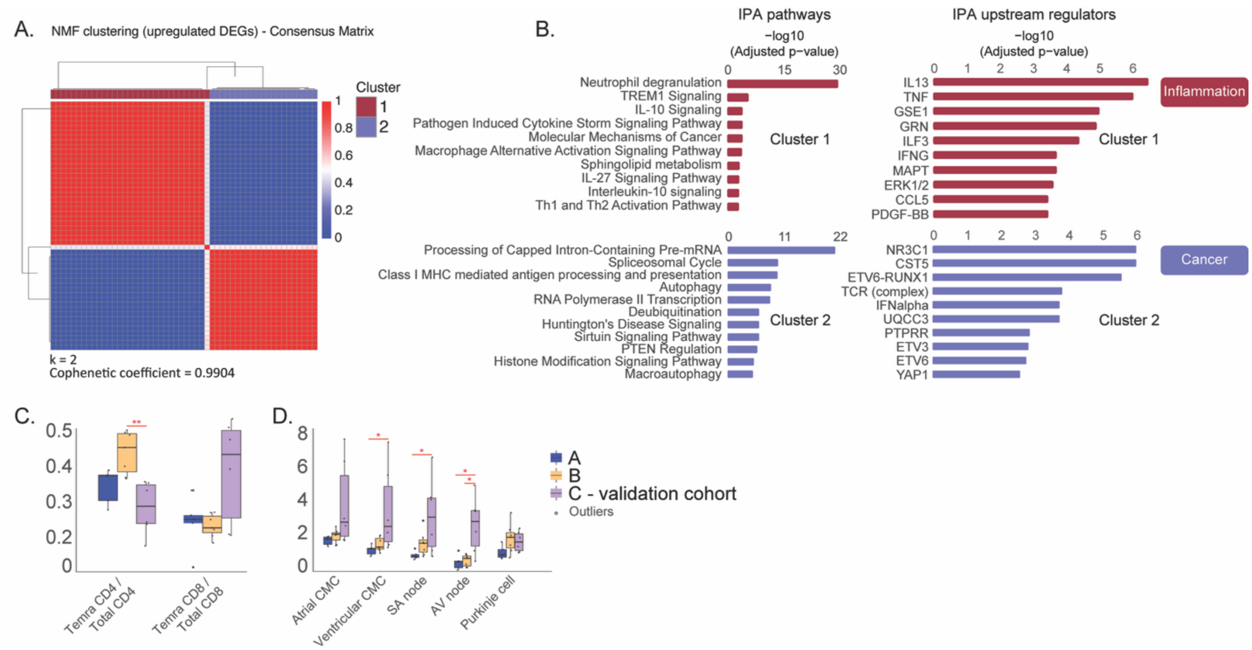

Supplemental Figure 2 – The plasma cf-mRNA profile differentiates two distinct patient populations and captures major disease pathways. A) Consensus matrix of non-negative matrix factorization (NMF) performed on only upregulated DEGs from ICI-cancer (n=22) vs. healthy controls (n=30). **B)** IPA pathway analysis with the top 10 enriched pathways and their corresponding upstream regulators of the two clusters identified by NMF. **C)** Boxplots comparing the signature score of Temra CD4+ and Temra CD8+ T cells as a proportion of the total CD4+ and CD8+ T cell populations in group C validation cohort compared to group A (n=5) and B (n=7) patients using cell type-specific gene panels from scRNAseq of circulating PBMCs from ICI-treated cancer patients (Zhu H et al., 2022) **D)** Cardiomyocyte subtypes signature scores in group C validation cohort (n=6) vs group A (n=5) and B (n=7) patients. *p < 0.05, **p < 0.01 by 2-tailed Mann-Whitney U test.

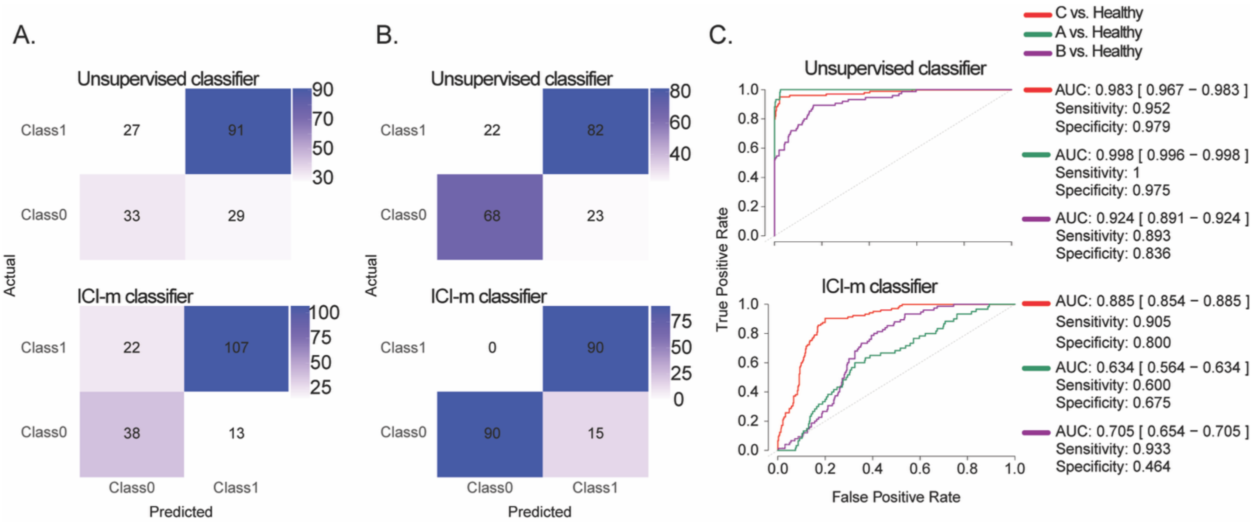

Supplemental Figure 3 – A deconvolution-derived diagnostic cf-mRNA gene panel can serve as candidate biomarkers for establishing the diagnosis of ICI-m. A) Confusion matrix of group C (n=10) vs. A (n=5) using the unsupervised gene panel (top) and ICI-m classifier (bottom). **B)** Confusion matrix of group C (n=10) vs. B (n=7) using the unsupervised gene panel (top) and ICI-m classifier (bottom). **C)** ROC curves and associated AUC, specificity, and sensitivity of C (red line, n=10), A (green line, n=5), or B (purple line, n=7) vs. healthy controls (n=30) using the unsupervised gene panel (top plot) or ICI-m classifier (bottom plot).
